# *PLOS Pathogens* 2017 Reviewer and Editorial Board Thank You

**DOI:** 10.1371/journal.ppat.1006958

**Published:** 2018-03-15

PLOS and the *PLOS Pathogens* team would like to express our appreciation for our associate editors, guest editors, and reviewers who contributed to the peer-review process this past year. We are indebted to our volunteers who generously give their time and expertise to thoroughly review research and advance Open Access. In 2017, *PLOS Pathogens* received the help of 226 Editorial Board members and 261 guest editors to curate 2,360 submissions. Along with the participation of 2,479 reviewers, we were able to publish 552 articles with meaningful and impactful results.

The names of our 2017 editors that handled submitted manuscripts appear in the Supporting Information as S1 Editor List and as S1 Guest Editor List. Our reviewers appear in the Supporting Information as S1 Reviewer List. With genuine gratitude, we thank you all for your dedicated support of *PLOS Pathogens* and our efforts to promote Open Science, thereby contributing to the scientific community as a whole. Thank you all.

## Supporting information

S1 Editor List Alphabetical list of 2017 *PLOS Pathogens* Editorial Board Members. (PDF)

S1 Guest Editor List Alphabetical list of 2017 *PLOS Pathogens* Guest Editors. (PDF)

S1 Reviewer List Alphabetical list of 2017 *PLOS Pathogens* peer reviewers. (PDF)

## References

[1] (2017) *PLOS Pathogens* 2016 Reviewer and Editorial Board Thank You. PLoS Pathog 13(3):e1006278 https://doi.org/10.1371/journal.ppat.100627.

[2] (2016) *PLOS Pathogens* 2015 Reviewer Thank You. PLoS Pathog 12(2):e1005497 https://doi.org/10.1371/journal.ppat.1005497.

[3] (2015) *PLOS Pathogens* 2014 Reviewer Thank You. PLoS Pathog 11(2):e1004748 https://doi.org/10.1371/journal.ppat.1004748.

