# Supplementary material for: PLOS Pathogens 2017 Reviewer and Editorial Board Thank You
Source: PLoS Pathog. 2018 Mar 15;14(3):e1006958. doi: 10.1371/journal.ppat.1006958 (PMC5854425; doi:10.1371/journal.ppat.1006958)
Supplement: S1 Editor List — (PDF) [file ppat.1006958.s001.pdf]

PLOS Pathogens would like to thank all those who served on the Editorial Board in 2017:

Umberto Agrimi
Christopher Aiken
Raul Andino
Alex Andrianopoulos
François Balloux
Jason C. Bartz
Christopher F. Basler
Andreas J. Baumler
Marcel A. Behr
Robert Belshaw
Chris A. Benedict
Nora J. Besansky
Debra E. Bessen
Stephen M. Beverley
Oliver Billker
Paul Birch
Michael J. Blackman
Steven R. Blanke
Helena Ingrid Boshoff
Kenneth A. Bradley
William J. Britt
Vern B. Carruthers
Sara Cherry
Ambrose Cheung
Jenifer Coburn
James J. Collins III
Brian K. Coombes
Isabelle Coppens
Laurent Coscoy
Leah E. Cowen
Carolyn B. Coyne
Robert A. Cramer
Bryan R. Cullen
Blossom Damania
Jeffery L. Dangl
Kirk W. Deitsch
Eric Y. Denkers
Isabelle Derré
Ronald C. Desrosiers
Michael S. Diamond
Savithramma P. Dinesh-Kumar
Shou-Wei Ding
Dirk P. Dittmer
Tamara L. Doering
Daniel C. Douek
Simon L. Dove
Sabine Ehrt
Nels C. Elde
Michael Emerman
Christian R. Engwerda
David T. Evans
Marta Feldmesser
Pinghui Feng
Neil M. Ferguson
Ana Fernandez-Sesma
Scott G. Filler
Erik K. Flemington
JoAnne L. Flynn
Sarah M. Fortune
Ron A. M. Fouchier
Daved H. Fremont
Klaus Früh
Michaela U. Gack
Sarah L. Gaffen
Denise A. Galloway
Andrea Gamarnik
Shou-Jiang Gao
Adolfo García-Sastre
William C. Gause
Ricardo T. Gazzinelli
Hui-Shan Guo
Kasturi Haldar
Alan R. Hauser
Thomas R. Hawn
Sheng Yang He
Patrick Hearing
Mark T. Heise
Joseph Heitman

Kent L. Hill
Deborah A. Hogan
Thomas J. Hope
David Horn
Lindsey Hutt-Fletcher
Francis Michael Jiggins
Patricia J. Johnson
Robert F. Kalejta
Yoshihiro Kawaoka
James Kazura
Kami Kim
Bruce S. Klein
Kimberly A. Kline
Laura J. Knoll
Theresa M. Koehler
Richard A. Koup
Hans-Georg Krausslich
Damian J. Krysan
Tomoko Kubori
Jens H. Kuhn
Richard J. Kuhn
Paul Francis Lambert
Jean Langhorne
Adam S. Lauring
Vincent T. Lee
Benhur Lee
David M. Lewinsohn
Paul M. Lieberman
Jeffrey Lifson
Xiaorong Lin
Paul D. Ling
James B. Lok
P'ng Loke
Richard Longnecker
Zhao-Qing Luo
Guangxiang George Luo
Wenbo Ma
Neil A. Mabbott
David Mackey
Michael H. Malim
Robin Charles May
Alison A. McBride
Grant McFadden
Elizabeth Ann McGraw
Craig Meyers
Timothy J. Mitchell
Aaron P. Mitchell
Edward Mitre
Edward Mocarski
Denise M. Monack
Ashlee V. Moses
Karen L. Mossman
Maria M. Mota
Ingrid Müller
Karl Münger
Eain A. Murphy
Peter D. Nagy
Xavier Nassif
Jay A. Nelson
Janko Nikolich-Zugich
Thomas B. Nutman
Audrey Ragan Odom
Carlos Javier Orihuela
Mary O'Riordan
Michael Otto
Jing-Hsiung James Ou
Peter Palese
Matthew R. Parsek
John T. Patton
Edward J. Pearce
Andrew Pekosz
Daniel R. Perez
Andreas Peschel
William A. Petri Jr.
Margaret A. Phillips
Dana J. Philpott
Ted C. Pierson
Alice Prince
Nancy Raab-Traub
Glenn F. Rall
Glenn Randall
Jason L. Rasgon
Laurie Read
Félix A. Rey
Charles M. Rice
Eleanor M. Riley
Christophe Ritzenthaler
Erle S. Robertson

Susan R. Ross
Craig R. Roy
David Sacks
Nina Reda Salama
Padmini Salgame
R. Jude Samulski
Andrea J. Sant
Christopher M. Sassetti
Karla J. F. Satchell
Connie S. Schmaljohn
David S. Schneider
Matthias Johannes Schnell
Erwin Schurr
H. Steven Seifert
Donald C. Sheppard
Barbara Sherry
Aleem Siddiqui
Luis J. Sigal
Anita Sil
Guido Silvestri
Joe Smith
Dominique Soldati-Favre
Samuel H. Speck
Mary M. Stevenson
Xin-Zhuan Su
Kanta Subbarao
Bill Sugden
Paul M. Sullam
Surachai Supattapone
Sankar Swaminathan
Ronald Swanstrom
Christoph Tang
Timothy L. Tellinghuisen
Volker Thiel
Paul G. Thomas
Bart Thomma
Greg Tiao
Guy Tran Van Nhieu
Alexandra Trkola
Renée M. Tsolis
Brett M. Tyler
Raphael H. Valdivia
Kenneth D. Vernick
Marco Vignuzzi
Christopher M. Walker
Aiming Wang
David Wang
David Weiss
Michael R. Wessels
David Westaway
Robert T. Wheeler
Sean P. J. Whelan
E. John Wherry
David L. Williams
Thomas A. Wynn
Jin-Rong Xu
Dario S. Zamboni
Gongyi Zhang
Z. Hong Zhou
Jian-Min Zhou
