# Supplementary material for: PLOS Pathogens 2017 Reviewer and Editorial Board Thank You
Source: PLoS Pathog. 2018 Mar 15;14(3):e1006958. doi: 10.1371/journal.ppat.1006958 (PMC5854425; doi:10.1371/journal.ppat.1006958)
Supplement: S1 Guest Editor List — (PDF) [file ppat.1006958.s002.pdf]

PLOS | PATHOGENS

*PLOS Pathogens* would like to thank all those who served as Guest Associate Editors in 2017:

Hervé Agaisse
J. Andrew Alspaugh
John D. Altman
Tim J. C. Anderson
Carlos F. Arias
Aravind Asokan
Walter J. Atwood
Simon A. Babayan
Megan Baldridge
Charles R. M. Bangham
Matthew F. Barber
Michael P. Barrett
Ralf Bartenschlager
Vivian Bellofatto
Cornelia C. Bergmann
Sonja Best
Roman Biek
David M. Bisaro
Samuel James Black
David Bloom
Jesse D. Bloom
Zbynek Bozdech
Alexandra Carolyn Brand
Paul J. Brindley
Igor Eric Brodsky
Wolfram Brune
Craig E. Cameron
Edward M. Campbell
James E. Cassat
Simon Cauchemez
Sumit K. Chanda
Alain Charbit
Hans H. Cheng
Peter Cherepanov
Gitta Coaker
Donald M. Coen
Jorn Coers
David J. Conway
Roland Cooper
James E. Crowe Jr.
Liwang Cui
Angus L. Dawe
Ralph Dean
Frank R. DeLeo
Neal A. DeLuca
Albert Descoteaux
Mahalia S. Desruisseaux
Darrell Desveaux
Adler Dillman
George Dimopoulos
Katie J. Doores
Christian Drosten
Siobain Duffy
Jeffrey D. Dvorin
Gregory D. Ebel
Richard Enelow
Luis Enjuanes
Lynn W. Enquist
David H. Evans
Michael Farzan
Zongdi Feng
Ralph Feuer
Luisa Figueiredo
Stefan Finke
Elizabeth P. B. Fontes
Vance G. Fowler
Matthew B. Frieman
Tom Gallagher
George F. Gao
Robert L. Garcea
Fernando Garcia-Arenal
Timothy G. Geary
Thomas Geisbert
Angie Gelli
Caroline A. Genco
Robert J. Gifford

Tim Wolf Gilberger
N. Louise Glass
Britt A. Glaunsinger
Felicia Goodrum
Andrea L. Graham
Urs F. Greber
Haitao Guo
Masanori Hatakeyama
Ekaterina E. Heldwein
Hartmut Hengel
Thomas Henry
Debroski R. Herbert
Volker Theo Heussler
Matthew K. Higgins
David A. Hildeman
Ann B. Hill
Tobias M. Hohl
Edward C. Holmes
Stacy M. Horner
Fajian Hou
Michael H. Hsieh
Jianming Hu
Linden T. Hu
Greg Hurst
Michael J. Imperiale
Roger W. Innes
Ken J. Ishii
Akiko Iwasaki
Ilse Jacobsen
Grant J. Jensen
Stipan Jonjic
Russell A. Jurenka
Jonathan Kagan
Isgouhi Kaloshian
Charu Kaushic
Paul M. Kaye
Kenneth M. Kaye
Nancy P. Keller
Shannon C. Kenney
Alexander A. Khromykh
Dennis H. Kim
Karla Kirkegaard
Sabra L. Klein
Julia Ruth Koehler
Alain Kohl
James B. Konopka
Anita Koshy
Florian Krammer
Peter J. Krause
Laurie Tate Krug
Michael Lagunoff
Scott M. Landfear
Michael Lattorff
Brian P. Lazzaro
Paul Lehner
David A. Leib
Philippe Lemey
Stanley M. Lemon
Deborah J. Lenschow
David E. Levy
Kui Li
James Lloyd-Smith
Carolina B. Lopez
Michael C. Lorenz
Anice C. Lowen
Harmit S. Malik
Ivan Marazzi
Richard J. Martin
Keith R. Matthews
Bruce A. McClane
Joan Mecsas
Xiang-Jin Meng
Lloyd S. Miller
Matthew S. Miller
Samuel I. Miller
Genevieve Milon
Harry L. T. Mobley
Joachim Morschhäuser
Jeremy C. Mottram
Matthew A. Mulvey
Philip M. Murphy
Thomas Naderer
Stuart J. D. Neil
Martha I. Nelson
Gabriele Neumann
Romolo Nonno

Christopher C. Norbury
Mairi C. Noverr
Martin Olivier
Eric Oswald
Annette Oxenius
Joseph S. Pagano
John S. L. Parker
James C. Paulson
Philip E. Pellett
Stanley Perlman
Sallie R. Permar
Nathan C. Peters
Philippe Plattet
Richard K. Plemper
Alexander Ploss
Christine Posavad
Marco Antonio Maximo Prado
Jayne Raper
Chad A. Rappleye
Laurent Rénia
Steven Riley
Amariliz Rivera
Michael D. Robek
Richard B. S. Roden
Robert Sabatini
Jeroen P. J. Saeij
Peter Sarnow
Sara L. Sawyer
Sebastian Schornack
Stacey Schultz-Cherry
W. Evan Secor
Michael F. Seidl
Murray E. Selkirk
Bert L. Semler
William M. Shafer
Libo Shan
Aimee Shen
Shin-Ru Shih
Sujan Shresta
Robert F. Siliciano
João Santana Silva
Photini Sinnis
David Skurnik
James M. Slauch
James R. Smiley
Christopher M. Snyder
Nahum Sonenberg
Abraham L. Sonenshein
Simona Stäger
Thilo Stehle
Adi Stern
Philip G. Stevenson
Daniel Neal Streblow
Lishan Su
Christopher S. Sullivan
Mehul Suthar
Nobuhiro Suzuki
Nicholas J. Talbot
Hengli Tang
Yizhi Jane Tao
Martin Taylor
David M. Tobin
Niraj H. Tolia
Emily R. Troemel
David C. Tscharke
Christian Tschudi
Kenneth L. Tyler
Jan van Kan
Ronald P. Van Rij
Ashley M. Vaughan
Jatin Mahesh Vyas
Yuanchao Wang
Nian Wang
Gary E. Ward
Wensheng Wei
Taiyun Wei
Joshua S. Weitz
Matthew D. Welch
Sandra K. Weller
Robert T. Wheeler
Marvin Whiteley
Lena Wilfert
Claus O. Wilke
Mark S. Wilson
Richard A. Wilson
Christiane E. Wobus

Matthew C. Wolfgang
Adrian J. Wolstenholme
Qingfa Wu
George S. Yap
Michael R. Yeaman
MinKyung Yi
John Yin
Andrew Yurochko
Allan J. Zajac
Zhi-Ming Zheng
Xueping Zhou
Fanxiu Zhu
Arturo Zychlinsky
