# Supplementary material for: PLOS Pathogens 2017 Reviewer and Editorial Board Thank You
Source: PLoS Pathog. 2018 Mar 15;14(3):e1006958. doi: 10.1371/journal.ppat.1006958 (PMC5854425; doi:10.1371/journal.ppat.1006958)
Supplement: S1 Reviewer List — (PDF) [file ppat.1006958.s003.pdf]

PLOS | PATHOGENS

PLOS Pathogens would like to thank all those who reviewed on behalf of the journal in 2017:

Pierre Abad
Ali Abdul-Sater
Jonathan Abraham
Margaret Ackerman
Alvaro Acosta-Serrano
Shelley Adamo
Joshua Adkins
Barbara Adler
Lynn Adler
Michael Adler
Toni Aebischer
Martin Aepfelbacher
Hervé Agaisse
Takashi Agui
Hector Aguilar
Sebastian Aguirre
Jesus Aguirre
Adriano Aguzzi
Ivan Ahel
Golo Ahlenstiel
Paul Ahlquist
Brian Ahmer
Jinwoo Ahn
Jin-Hyun Ahn
Tero Ahola
Sunil Ahuja
Christopher Aiken
Gillian Air
Bungo Akiyoshi
Klaus Aktories
Markus Albert
Jan Albert
Randy Albrecht
Jose Alcami
John Alcorn
Holly Algood
Samuel Alizon
David Alland
Rachel Allen
Judith Allen
J. Alspaugh
Nihal Altan-Bonnet
Galit Alter
Marcus Altfeld
Craig Altier
John Altman
Neal Alto
Xavier Alvarez
Gaya Amarasinghe
Zandrea Ambrose
Amal Amer
Manuel Amieva
Maria Joao Amorim
Larry Anderson
Jeffrey Anderson
David Andes
Warrison Andrade
Olivier Andréoletti
Norma Andrews
Helene Andrews-Polymenis
Elliot Androphy
Simon Anthony
J. Antonio del Rio
Cristian Apetrei
Richard Apps
Ravit Arav-Boger
Laurence Arbibe
Darius Armstrong-James
Dawn Arnold
Michelle Arnold
Damien Arnoult
James Arthos
Sassan Asgari
David Askew
Georgia Atella
Geoffrey Attardo

Walter Atwood
Susann Auer
Victoria Auerbuch
Laurent Aussel
Frédéric Auvray
Steffen Backert
Marija Backovic
Andre Bafica
Justin Bahl
Xiyuan Bai
Lauren Bakaletz
Barbara Baker
Siddharth Balachandran
Jonathan Ball
Jimmy Ballard
David Baltrus
Bruce Banfield
Mark Banfield
Jay Bangs
Lawrence Banks
Shweta Bansal
Ashley Banyard
Benoît Barbeau
Matthew Barber
Daniel Barber
Wendy Barclay
Amy K. Barczak
Stephen Barenkamp
Francis Barin
Christian Baron
Frédéric Barras
Daniel Barreda
Luis Barreiro
Alan Barrett
Peter Barry
Rebecca Bart
Ralf Bartenschlager
Sina Bartfeld
Lyric Bartholomay
David Barton
Ilia Baskakov
Marek Basler
Joseph Bass
Diane Bassham
Paul Bates
Simon Baxter
Wibke Bayer
Arnold Bayer
Quentin Bazot
Rupert Beale
Clifford Beall
Michael Beard
J. David Beckham
Sammy Bedoui
Robin Beech
James Beeson
Samuel Behar
Youssef Belkhadir
George Belov
Graham Belsham
Monsef Benkirane
Richard Bennett
Rance Berg
Jeffrey Bergelson
Tessa Bergsbaken
Andreas Bergthaler
Vincent Beringue
Arnold Berk
Ben Berkhout
Judith Berman
Luiz Bermudez
Nicole Bernard
Kristen Bernard
David Bernlohr
Patrick Bertolino
Sonja Best
Charles Bevins
Sumita Bhaduri-McIntosh
Alok Bhattacharya
Tanmoy Bhattacharya
Deepta Bhattacharya
Mrinal Bhattacharyya
David Bhella
James Bina
Robert Binder
Brian Bird

Christine Biron
Kate Bishop
Partha Biswas
Thomas Bjarnsholt
Niklas Björkström
Dieter Blaas
Jason Blackard
Ira Blader
Joshua Blakeslee
Joel Blankson
Catherine Blish
James Bliska
Joseph Bliss
Vivian Blok
Danielle Blondel
David Bloom
Debby Bogaert
Dusan Bogunovic
Melvin Bolton
Jennifer Bomberger
Ivo Boneca
Maciej Boni
Robert Bonomo
Mattia Bonsignori
Adrianus Boon
Andre Boonstra
Stephanie Booth
Seth Bordenstein
Eli Boritz
Katherine Borkovich
Frederik Börnke
Eli Borrego
Berend Jan Bosch
Irene Bosch
Michael Boshart
Steven Bosinger
Cyrille Botté
Jason Botten
Fadila Bouamr
Michael Bouchard
Thierry Boulinier
Freddy Boutrot
Dawn Bowdish
David Bowen
Jeffrey Boyd
Jon Boyle
Michelle Boyle
Joel Bozue
Patricia Bozza
Steven Bradfute
Peter Bradley
L. Jeannine Brady
Oliver Brady
Axel Brakhage
Marc Bramkamp
Christian Brander
Curtis Brandt
Aaron Brault
Stephen Braun
Sigal Braun Miyara
Miriam Braunstein
Jay Bream
Jason Brenchley
Richard Brennan
S. Bressanelli
James Brewer
Volker Briken
David Briles
Melanie Brinkmann
William J. Britt
Warwick Britton
Nichole Broderick
Igor Brodsky
Barbara Broeker
Jürgen Brojatsch
Barbara Bröker
Frank Brombacher
Denise Bronner
Chris Brooke
Roland Brosch
Candice M. Brown
Eric Brown
Gordon Brown
Jeremy Brown
Edward Browne
Angela Brueggemann

Zabrina Brumme
John Brummel
Wolfram Brune
Volker Bruss
Shilpa Buch
Nicolas Buchon
Amy Buck
Frederick Buckner
Mauricio Budini
Alexander Bukreyev
Michael Bukrinsky
Esther Bullitt
Tessa Burch-Smith
Tricia Burdo
Adam Burgener
Barbara Burleigh
Oscar Burrone
Lori Burrows
Dennis Burton
Kathryn Bushley
Frederic Bushman
Víctor Bustamante
Mariana Byndloss
Siddappa Byrareddy
Ken Cadwell
Qiliang Cai
Yingyun Cai
Judy Callis
Mark Cameron
Jennifer Cameron
Gabriella Campadelli-Fiume
Lee Ann Campbell
David Campbell
Edward Campbell
Kenneth Campellone
Samuel Campos
Bruno Canard
Gerard Cangelosi
Edouard Cantin
Patrizia Caposio
Rey Carabeo
Nicholas Carbonetti
Jan Carette
Andrea Carfi
John Carr
Paola Carrillo-Bustamante
Mark Carrington
Mary Carrington
Arturo Casadevall
Jim Casanova
Eric Cascales
James Cassat
Francis Castellino
Joaquín Castilla
Marta Catalfamo
Isabella Cattadori
Byron Caughey
Demian Cazalla
Lisa Cazares
Lynette Cegelski
Jean Celli
Anna Cereseto
Ethel Cesarman
Kris Chadee
Ann Chahroudi
Arup Chakraborty
Henry Chambers
Georgios Chamilos
Gary Chan
Kartik Chandran
Guillaume Chanfreau
Kyong-Mi Chang
Yuan Chang
Theresa Chang
Jeff Chang
Nora Chapman
Mounira Chelbi-Alix
Linda Chelico
Mei-Ru Chen
Bing Chen
Xiaojiang Chen
Xuewei Chen
Yuting Chen
Gong-You Chen
Ding Chen
Mingzhou Chen

Benjamin Chen
Alan Cheng
Gong Cheng
Genhong Cheng
Cecilia Cheng-Mayer
Peter Cherepanov
Chetan Chitnis
Christopher Chiu
Shusin Chng
Nam-Hyuk Cho
James Chodosh
Sang Ho Choi
Nicolas Chomont
Heng Chooi
Neil Christensen
Rebecca Christofferson
Heather Christofk
George Christophides
Ewa Chrostek
Hiutung Chu
Tae-Wook Chun
Luka Čičin-Šain
Andrea Cimarelli
Gino Cingolani
Mara Cirone
Angela Ciuffi
Marcus Clark
David Clarke
Thomas Clarke
Jean-Michel Claverie
Christine Clayton
Rollie Clem
Janice Clements
Anna Cliffe
Cevayir Coban
Brian Cobb
Sarah Cobey
Ian Cockburn
Donald Coen
Jorn Coers
John Coffin
Frederick Cohan
Jeffrey Cohen
Eric Cohen
Taylor Cohen
Kathleen Collins
Steven Collins
Alan Collmer
Arnaldo Colombo
Marco Colonna
Tonya Colpitts
Valery Combes
Alex Compton
Richard Condit
Nicholas Conrad
Carlos Contreras-Martel
Klaus Conzelman
Klaus Conzelmann
David Cook
Lucy Cook
Brian Cooke
Arik Cooper
Davide Corti
Jenny Cory
Sergio Costa Oliveira
Peggy Cotter
Sheena Cotter
Matthew Cotton
Fasseli Coulibaly
Sarah Coulthurst
Kevin Couper
Mathieu Coureuil
Anna Coussens
Bob Coutts
Carolyn Coyne
Alister Craig
Darren Creek
Max Crispin
Peter Crompton
John Cronan
George Cross
Alan Cross
Robert Cross
Jimmy Crott
Nicholas Croucher
Tony Crowther

Karine Crozat
Richard Culleton
Fiona Culley
James Cunningham
Anthony Cunningham
Adam Cunningham
Tom Cupedo
Stephen Curry
Jason Cyster
Yasin F. Dagdas
Wassim Daher
Wei Dai
Lisa Daley-Bauer
Marc Dalod
John Dalton
Satya Dandekar
Pranav Danthi
Saumitra Das
Sandip Datta
Miles Davenport
Robert Davey
Stephen Davies
Jean-Luc Davignon
Kimberly Davis
Richard Davis
Andrew Davison
Suzanne Dawid
Lisa Dawson
William Dawson
Brad Day
Cheryl Day
Janice de Almeida Engler
Miranda De Graaf
Christopher de Graffenried
Victor De Gruttola
Cornelis de Haan
Ronnie de Jonge
Harry De Koning
Tania de Koning-Ward
Chris de Koster
Adela de la Campa
Leonardo De La Fuente
Juan C. de la Torre
Giulio De Leo
Hilde de Reuse
Aravinda de Silva
Emmie de Wit
Marcel de Zoete
Samuel Dean
Erik Debler
Herbert De'Broski
Zeger Debyser
Thomas DeCoursey
Steven Deeks
George Deepe Jr.
Victor DeFilippis
Ala-Eddine Deghmane
Christoph Dehio
Rosa Maria del Angel
Luis Del Valle
Pierre-Marc Delaux
Henri-Jacques Delecluse
Mónica Delgado
Francis Delpeyroux
Neal DeLuca
Nicolas Demaurex
Jane Deng
Liang Deng
Yizhen Deng
Mark Denison
Timothy Denning
Paul Denton
Keith Derbyshire
Cynthia Derdeyn
Vojo Deretic
Petra Dersch
Prashant Desai
Sanjay Desai
Albert Descoteaux
Laurent Deslandes
Delphine Destoumieux-Garzon
Darrell Desveaux
Ludovic Desvignes
Corrella Detweiler
Rebekah DeVinney
Michele Di Mascio

Antonio Di Pietro
Gill Diamond
Arturo Diaz
Martin Dickman
Paul Digard
Peter Digennaro
Joseph Dillard
Adler Dillman
Daniel Dimaio
George Dimopoulos
Rhoel Dinglasan
Bismarck Dinko
Marc Dionne
Richard Dipaolo
Colette Dissous
Luciano Ditacchio
Dirk Dittmer
Ulf Dittmer
Maziar Divangahi
Richard Dix
Roberto Docampo
David Dockrell
Peter Dodds
Gunther Doehlemann
Christian Doerig
Robert Doms
Anna Dongari-Bagtzoglou
Christl Donnelly
Russell Doolittle
John Doorbar
Katie Doores
Kelly Doran
Sarah D'Orazio
Charles Dorman
Philip Dormitzer
John Dow
Shaynoor Dramsi
Marlène Dreux
Jan Felix Drexler
Ingo Drexler
Robert Drillien
Heidi Drummer
Rebecca Drummond
Cheng-Guo Duan
Jean Dubuisson
George Dubyak
Robert Duda
Gytis Dudas
Jaquelin Dudley
Guillaume Dumenil
Stephen Duncan
Christine Dunham
David Dunigan
Joan Durbin
Michael Duszenko
Rebecca Dutch
Jeffrey Dvorin
Jonathan Dworkin
Jose Echenique
Karen Edelblum
Mira Edgerton
Frank Edlich
Michael Edwards
Stacey Efstathiou
Martin Egan
Dirk Eggink
Ian Ehrenreich
Laurence Eisenlohr
Britta Eiz-Vesper
John Elder
Ioannis Eleftherianos
Jean-François Eléouët
Ayman El-Guindy
Paul Elkington
Ali Ellebedy
Craig Ellermeier
Stephane Emiliani
Richard Enelow
Joanne Engel
Alan Engelman
David Engman
Ylva Engstrom
Patrik Engström
Lynn Enquist
Armin Ensser
Ghigo Eric

Silvio Erler
Joel Ernst
Jonatan Ersching
Audrey Esclatine
José Esté
Lucie Etienne
Tom Evans
David Evans
Matthew Evans
Teresa Evering
David Everly
Katherine Excoffon
Robert Fagan
Keke Fairfax
Erik Falck-Pedersen
Bryce Falk
Ann Fallon
Jing Fan
Bentley Fane
Chuck Farah
Paul J. Farrell
John Farrow
Ariberto Fassati
Herman Favoreel
Michael Federle
Zhangjun Fei
Ten Feizi
Mario Feldman
Simon Fellous
Youjun Feng
Zongdi Feng
Michael Ferdig
Heather Ferguson
Michael Ferguson
Dominique Ferrandon
Juan Ferré
Martin Ferris
Paul Fidel
David Fidock
Kenneth Fields
Andrés Finzi
Nicole Fischer
Louis Flamand
J. Bert Flanegan
Suzanne Fleiszig
Courtney Fletcher
S. Flint
Luise Florin
Ervin Fodor
Kelly Folkers
Denise Fonseca
Thierry Fontaine
Emily Ford
Naomi Forrester
Donald Forthal
G. Foster
Keith Fowke
Howard Fox
James Fox
Ellen Foxman
Tatiana Fraga
Genoveffa Franchini
Ivo Francischetti
Dara Frank
Gad Frankel
Alexander Franz
Iain Fraser
Eric Freed
Lisa Frenkel
Manuel Fresno
Eva Frickel
Harvey Friedman
Thomas Friedrich
Matthew Frieman
Niels Frimodt-Moller
Friedrich Frischknecht
Jörg Fritz
Serge Fuchs
Robert Fujinami
Catarina Gadelha
Tom Gallagher
Awen Gallimore
Jean-Luc Gallois
David Gally
Ohad Gal-Mor
Amit Gal-On

Don Gammon
Rajesh Gandhi
Chengjiang Gao
Robert Garcea
J. Victor Garcia
Francisco García-del Portillo
Hernan Garcia-Ruiz
Nisha Garg
Victoriano Garre
Danielle Garsin
Beth Garvy
Yves Gaudin
Julie Gavard
Nicholas Gay
Ricardo Gazzinelli
Peng Ge
Timothy Geary
Steven Geary
Adam Geballe
Adam Gehring
Teunis Geijtenbeek
Thomas Geisbert
Brian Geisbrecht
Angie Gelli
Howard Gendelman
Elke Genersch
Martin Gengenbacher
Stephane Genin
Nicole Gerardo
Gisa Gerold
Andrew Gewirtz
Benjamin Gewurz
Elodie Ghedin
Frank Gheradini
Godelieve Gheysen
Partho Ghosh
Chou-Zen Giam
Sara Gianella
Wendy Gibson
Francis Gigliotti
Tim Gilberger
Peter Gilbert
Sarjeet Gill
Laurent Gillet
Nicolas Gillet
Eleanor Gilroy
Paul Gilson
Michael Ginger
Stephen Girardin
Patrick Giraudoux
Tor Gjøen
Pierre Gladieux
N. Louise Glass
Markus Glatzel
Britt Glaunsinger
Jane Glazebrook
Cynthia Gleason
Paul Goepfert
Stephen Goff
Jon Goguen
Joanna Goldberg
Marcia Goldberg
Daniel Goldberg
Pascal Goldschmidt-Clermont
Thaddeus G. Golos
Tatyana Golovkina
Mark Gomelsky
Maria Gomes-Solecki
James Gomez
Leslie Goo
Michael Goodin
Stephen Goodwin
Nilu Goonetilleke
Alexander Gorbalenya
Jean-Pierre Gorvel
Irene Görzer
Susan Gottesman
Heinrich Gottlinger
Marcelo Gottschalk
Friedrich Götz
Sven Gould
Neil Gow
D. Channe Gowda
Sheila V. Graham
Stephen Graham
John Grainger

Arash Grakoui
Christophe Grangeasse
Warwick Grant
Urs Greber
Harry Greenberg
Robert Greenberg
Justin Greene
Mallary Greenlee-Wacker
Richard Grencis
Giorgio Gribaudo
Scott Grieshaber
Stephen Griffin
Diane Griffin
Matthias Gromeier
Chuck Grose
Alan Grossman
Christoph Grundner
Marc-Jan Gubbels
Bruno Guigas
Nancy Guillen
Keith Gull
Suryaram Gummuluru
John Gunn
Arthur Gunzl
Ju-Tao Guo
Haitao Guo
Nishith Gupta
Ravindra Gupta
Kurt Gustin
Maximiliano Gutierrez
Bart Haagmans
Rainer Haas
Albert Haas
Hubertus Haas
Ted Hackstadt
Andrew Haddow
Maria Hadjifrangiskou
Julius Clemence Hafalla
Susan Hafenstein
Anders Hafrén
Young Hahn
Nancy Haigwood
Hillel Haim
Stephen Hajduk
Mohamed Hakimi
Ben Hale
Rebecca Hall
Cherisse Hall
Elissa Hallem
Otto Haller
Luanne Hall-Stoodley
Scott Halstead
Marc Halushka
Shinjiro Hamano
Jessica Hamerman
Tansy Hammarton
Brian Hammer
Gianna Hammer
Wolfgang Hammerschmidt
Iqbal Hamza
Lars Hangartner
Thomas Hannan
Diana Hansen
Ashraful Haque
Wolf-Dietrich Hardt
Philip Hardwidge
Richard Hardy
Edward Harhaj
Billy Harnett
Melanie Harriff
Steven Harris
Mark Harris
Eva Harris
David Harris
Elizabeth Hartland
Eric Harvill
Kim Hasenkrug
Hassan Hashimi
Masanori Hatakeyama
Norman Haughey
Guillaume Hautbergue
John Hawdon
Yoku Hayakawa
Tetsuya Hayashi
Richard Hayward
Yan He

Chuan He
Biao He
Bin He
Cari Hearn
Nicholas Heaton
Stephen Hedrick
P. Scott Hefty
Johannes Hegemann
Adrian Hehl
Mohammad Heidari
Ernest Heimsath Jr.
Manfred Heinlein
Zdenek Hel
Sophie Helaine
Ekaterina Heldwein
Robert Hendricks
Adriano Henriques
Thomas Henry
Michael Hensel
Scott Hensley
Katherine Henzler-Wildman
Jussi Hepojoki
Debroski Herbert
Allen Herbst
Michael Herfs
Sander Herfst
Ruben Hernandez-Alcoceba
Elisabeth Herniou
Betsy Herold
Tomer Hertz
Volker Heussler
James Hewitson
Hubert Hilbi
David Hildeman
Rolf Hilgenfeld
Darryl Hill
Andrew Hill
Bradley Hillman
Julian Hillyer
B. Joseph Hinnebusch
Jay Hinton
Catarina Hioe
Vanessa Hirsch
Alec Hirsch
John Hiscott
Amy Hise
Simon Ho
Ya-Chi Ho
May Ho
Karin Hochrainer
Ary Hoffmann
Alexander Hoffmann
Daniel Hofius
Daniel Hoft
Deborah Hogan
Brenda Hogue
Tobias Hohl
Lindy Holden-Dye
Edward Holmes
Christoph Hölscher
Yeonchul Hong
Nigel Hooper
Edward Hoover
Felix Hoppe-Seyler
Stacy Horner
Tiffany Horng
William Horsnell
Fajian Hou
Charles Howe
Michael Hsieh
Wei-Shau Hu
Jianming Hu
Patrick Hu
Guochang Hu
Ke Hu
I-Chueh Huang
Huan Huang
Yihua Huang
Johannes Hübner
Amy Hudson
Jaime Huerta-Cepas
Gary Huffnagle
Stephen Hughes
Joseph Hughes
Christina Hull
Scott Hultgren

Dan Hultmark
Richard Humber
Ian Humphreys
David Hunstad
Peter Hunt
Henry Hunt
Craig Hunter
Christopher Hunter
Christopher Huston
Anna Huttenlocher
Seungmin Hwang
Kevin Hybiske
Alexandra Ibáñez-Escribano
Tetsuro Ikegami
Masaki Imai
James Imlay
Jean-Luc Imler
Michael Imperiale
Molly Ingersoll
Alyssa Ingmundson
Y. Tony Ip
Keith Ireton
Stuart Isaacs
Masanori Isogawa
Stanimir Ivanov
Akiko Iwasaki
Yoshihiro Izumiya
Angelo Izzo
Joseph Jackson
William Jackson
Enno Jacobs
William Jacobs
Kim Jacobson
Rakesh Jain
Sanjay Jain
Leo James
Anthony James
Eric Jan
Dragana Jankovic
Edward Janoff
Jonathan Jantsch
Christian Janzen
Armando Jardim
Kate Jeffrey
Peter Jensen
Jong-Seong Jeon
Travis Jewett
Yulin Jia
Baoming Jiang
Taijiao Jiang
Daohong Jiang
Lubin Jiang
Dong-Yan Jin
Ling Jin
Peng Jin
Rongsheng Jin
Eric Johannsen
Chandy John
Jack Johnson
R. Paul Johnson
Reed Johnson
Karyn Johnson
Welkin Johnson
Simon Johnston
Nicole Joller
Clare Jolly
Kristina Jonas
Malcolm Jones
Rheinallt Jones
John Jones
Lucy Jones
Christopher Jones
Clinton Jones
Ilse Jongerius
Stipan Jonjic
Kelly Jordan-Sciutto
Peter Jorth
Sarah Joseph
Wolfgang G. Junger
Juan Luis Jurat-Fuentes
Aras Kadioglu
David Kadosh
Jonathan Kagan
Barbara Kahl
William Kaiser
Kriton Kalantidis

Rob Kalejta
Daniel Kalman
Shaden Kamhawi
Sophien Kamoun
Satoko Kanematsu
Michael Kann
Mari Kannagi
Daniel Kaplan
John Karijolich
Fatah Kashanchi
Dennis Kasper
Peter Katsikis
Richard Katz
Daniel Kaufmann
Amitinder Kaur
Yasushi Kawaguchi
Yoshihiro Kawaoka
Paul Kaye
Kenneth Kaye
Barbara Kazmierczak
Joseph Keane
Dean Kedes
Ross Kedl
Brandon Keele
Ralph Kehlenbach
Thomas Kehl-Fie
Harald Keller
Nancy Keller
John Kelly
Alyson Kelvin
Bettina Kempkes
Scott Kennedy
Shannon Kenney
Stephen Kent
Florian Kern
Vineet Kewalramani
Nemat Keyhani
Shabaana Khader
Chang Hyun Khang
Kamal Khanna
Alexander Khromykh
Margaret Kielian
Hans-Peter Kiem
Hirokazu Kimura
Makoto Kimura
Paul Kinchington
Christine King
Kayla King
Robert Kingsley
Frank Kirchhoff
Natalia Kirienko
Karla Kirkegaard
Laura Kirkman
Kiyoshi Kita
Scott Kitchen
Tohru Kiyono
Morten Kjos
Nichole Klatt
Robyn Klein
Sabra Klein
Paul Klenerman
Kimberly Kline
Michele Klingbeil
Aloysius Klingelhutz
Don Klinkenberg
Karl Klose
Oren Kobiler
Lester Kobzik
Stefan Kochanek
Julia Koehler
David Koelle
Karl-Heinz Kogel
Andrew Koh
Alain Kohl
Satoshi Koike
Nikolay Kolev
Jay Kolls
Dennis Kolson
Qingzhong Kong
James Konopka
Sophia Koo
Manfred Kopf
Alexei Korennykh
Thomas Korn
Hardy Kornfeld
Daniel Kornitzer

Poonum Korpe
Sergei Kosakovsky Pond
Anita Koshy
Konstantin Kousoulas
Susan Koval
Pavel Kovarik
Yoshio Koyanagi
Florian Krammer
Igor Kramnik
Philip Kranzusch
Sven Krappmann
Simon Krattinger
Philip Krause
Ute Krengel
Jan Kreuze
Thomas Krey
James Kronstad
Laurie Krug
Eric Krukonis
Claude Krummenacher
Yasuyuki Kubo
Adam Kucharski
Meta Kuehn
Carol Kumamoto
Kota Arun Kumar
Sanjai Kumar
Ashok Kumar
Stefan Kunz
Rei-Lin Kuo
Marcelo Kuroda
Jonathan Kurtis
Olaf Kutsch
Mamuka Kvaratskhelia
Brian Kvitko
Terry Kwok
Douglas Kwon
Peter Kwong
D. Borden Lacy
Michael Laessig
Frank Lafont
Michael Lagunoff
Thomas Lahaye
Lou Laimins
Seema Lakdawala
Jean-François Laliberté
Daniel Lamarre
Tracey Lamb
Louis Lambrechts
Richard Lamont
Brigitte Lamy
Ke Lan
Nathaniel Landau
Santo Landolfo
Roland Lang
Marc-André Langlois
Ryan Langlois
Antonio Lanzavecchia
Gérald Larrouy-Maumus
Corinne Lasmezas
Jean-Paul Latge
Wyndham Lathem
Chris Lauber
Georg Lauer
Renia Laurent
Grégoire Lauvau
Dimitri Lavillette
Mansun Law
Beth Lazazzera
Helen Lazear
Brian Lazzaro
Roger Le Grand
Karine Le Roch
Frederique Le Roux
Chih-Hao Lee
Yin-Won Lee
Joon-Hee Lee Lee
Yong-Hwan Lee
Kevin Legge
Giuseppe Legname
Paul Lehner
Salomé Leibundgut-Landmann
Cynthia Leifer
Isabelle Lemasson
Niels Lemmermann
Wayne Lencer
Laurel Lenz

John Leong
Gebhard Leopoldo
Keith Leppard
Julien Lescar
Cammie Lesser
François Leulier
Ka Yin Leung
Nicolas Leveque
Stuart Levitz
Massimo Levrero
Sharon Lewin
Peter Lewis
Zachary Lewis
George Lewis
Xin Li
Lin-Xi Li
Ganwu Li
Liming Li
Jianrong Li
Wenhui Li
Bibo Li
Ziyin Li
Dianfan Li
Kui Li
Zhenghe Li
Xiao-Dong Li
Chengyu Liang
Chen Liang
Qiming Liang
Stephen Libby
Paul Liberman
Susan Liebman
Graham Lieschke
Jean Lim
Dominique Limoli
Rongtuan Lin
Zhen Lin
Xin Lin
Rafael Linden
Brett Lindenbach
Stefan Linder
Scott Lindner
Steven Lindow
Robin Lindsay
Miia Lindström
Jaisri Lingappa
Michail Lionakis
Howard Lipton
John Lipuma
Vladimir Litvak
Yael Litvak
Haoping Liu
Jun Liu
Jinhua Liu
Yule Liu
George Liu
Huiquan Liu
Fenyong Liu
James Lloyd-Smith
Shawn Lockhart
Melissa Lodoen
Martin Loechelt
Volker Lohmann
Philippe Loiseau
James Lok
James Lokensgard
Kristen Lokken
Ben Longdon
Yueh-Ming Loo
Ulisses Lopes
Susana Lopez
Carolina Lopez
Nora Lopez
Daniel López
Jose L. Lopez-Ribot
Constantino López-Macías
Juan Jose Lopez-Moya
Michael Lorenz
Marcelo Lorenzo
Alex Loukas
Sebastian Lourido
Crystal Loving
Anice Lowen
Rui Lu
Chun Lu
Mengji Lu

Shan Lu
Ling Lu
Jeremy Luban
Tim Lucas
Carsten Lüder
Stephan Ludwig
Carsten G. Lueder
Micah Luftig
Nicholas Lukacs
Julius Lukes
Slawomir Lukomski
Jennifer Lund
C. Patrick Lusk
Joe Lutkenhaus
Hinh Ly
Rebecca Lynch
Li Jun Ma
Jiyan Ma
Zhonghua Ma
Andrew MacDonald
Carlos Machado
Alberto Macho
Jason Mackenzie
Erich Mackow
James MacRae
Hiten Madhani
Cressida Madigan
Piet Maes
Markus Maeurer
Dixie Mager
Melissa Maginnis
Ravi Mahalingam
Suresh Mahalingam
Renaud Mahieux
Martin Maiden
Taronna Maines
Mala Maini
Gunnar Mair
Vladimir Majerciak
Johnson Mak
Kristiina Mäkinen
Shinji Makino
Krishnamurthy Malathi
Frank Maldarelli
Carolyn Malmstrom
Kevin Maloy
Nicolas Manel
Balaji Manicassamy
Colin Manoil
Susanna Manrubia
Benoit Marçais
Joseph Marcotrigiano
Ingrid Marcq
David Margolis
Chelsea Marie
Brian Mark
Thomas Marlovits
João Marques
Joao Marques
Adriana Marques
Mark Marsh
Matthias Marti
Darren Martin
Richard Martin
Carlos Martin
Javier Martin
Estelle Martin
Juan Martinez
Jennifer Martinez
Joaquin Martinez
Luis Martinez-Sobrido
Mauricio Martins
Preston Marx
Shin-Yi Lee Marzano
Andrea Marzi
Ruth Massey
Paul Masters
Maria Masucci
Candace Mathiason
Masao Matsuoka
Yoshiharu Matsuura
Keith Matthews
Stephen Matthews
Wendy Maury
Karen Maxwell
Katrin Mayer-Barber

Evaristus Mbanefo
Shonna McBride
Laura-Isobel McCall
Alex McCarthy
Malcolm McConville
John McCormick
Craig McCormick
Peter McCourt
Laura Mccoy
Richard McCulloch
Joseph McCune
Adrian McDermott
Kathleen McDonough
John McDowell
Anita McElroy
Geoffrey McFadden
James McGettigan
Kevin McIver
Derek McKay
Debbie McKenzie
James McLachlan
Paul McLaren
Jason McLellan
Rachel McLoughlin
Conor McMeniman
Andrew McMichael
Joseph McPhee
Stephen McSorley
Michael McVoy
David Meckes
Joan Mecsas
Andrew Mehle
Rahim Mehrabi
Borna Mehrad
Jeffery Meier
Annemarie Meijer
Markus Meissner
Anat Melamed
Peter Melby
Jose Melero
Gregory Melikyan
John Mellors
Espen Melum
Vineet Menachery
Alfredo Menendez
Andrew Mercer
Jason Mercer
Doron Merkler
Janet Mertz
Steven Meshnick
Ilhem Messaoudi
William Messer
Dennis Metzger
Eliane Meurs
José Meyer-Fernandes
Jun Miao
Thomas Michiels
Martin Middendorf
Nicole Mideo
Sandra Milasta
Jens Milbradt
Virginia Miller
Wolfgang Miller
William Miller
Glenn Millhauser
Hitomi Mimuro
Jonathan Miner
Chris Minion
Prashant Mishra
Thomas Mitchell
Yorgo Modis
John Modlin
Christopher Mody
Ian Mohr
Sachel Mok
Soren Molin
Ian Molineux
Malcolm Molyneux
Claudia Monari
Vicente Monedero
Véronique Monnet
Xavier Montagutelli
Mauricio Montal
David Montefiori
M. Moody
Cary Moody

Shannon Moonah
Penny Moore
Patrick Moore
Martin Moore
Margo Moore
Nathaniel Moorman
Darius Moradpour
Trevor Moraes
Rodrigo Morales
Thomas Moran
Dana Mordue
Luciano Moreira
Jean-Benoît Morel
Silvia Moreno
Yasuko Mori
Hiromitsu Moriyama
Koki Morizono
Craig Morrell
Lynn Morris
Thomas Morrison
Donald Morrison
Naomi Morrissette
Brooke Morriswood
Joachim Morschhäuser
Renato Mortara
Matthew Moscou
Gregory Moseley
George Mosialos
Jose Maria Alvarez Mosig
Laurent Mosnier
Paul Moss
Bernard Moss
Walter Moss
David Mosser
Serge Mostowy
Walther Mothes
Jeremy Mottram
Joseph Mougous
Sophie Mouillet-Richard
Andrew Mouland
Nissin Moussatche
Scott Moye-Rowley
Gaia Muallem
Anne Mueller
Scott Mueller
Nicolas Mueller
Elke Mühlberger
Shaeri Mukherjee
Amitabha Mukhopadhyay
Suchetana Mukhopadhyay
Yves Muller
Michaela Müller-Trutwin
Matthew Mulvey
Joshua Munger
Vincent Munster
Takeshi Murata
Pablo Murcia
Susan Murray
Ben Murrell
Nick Muzyczka
Peter Myler
Kevin Myles
Indira Mysorekar
Raffael Nachbagauer
Meera Nair
Shigetou Namba
Asuka Nanbo
Michael Nassal
Avindra Nath
Baibaswata Nayak
Daniel Neafsey
Richard Neher
Stuart Neil
Daniel Neill
Sergei Nekhai
David Nelson
Martha Nelson
Christian Nelson
Glen Nemerow
Donna Neumann
Michael Nevels
Cedric Neveu
Dawn Newcomb
Irene Newton
Olivier Neyrolles
Lisa Ng

Wai-Leung Ng
Sydney Nguyen
Dao Nguyen
Timothy Nice
Anthony Nicola
Kirsten Nielsen
Hartmut Niemann
Noriyuki Nishida
Marc Nishimura
Yorihiro Nishimura
Douglas Nixon
Victor Nizet
Angela Nobbs
Laurent Noel
Nicholas Noinaj
Christopher Norbury
Mairi Noverr
Tomoyoshi Nozaki
Sean-Paul Nuccio
Jack Nunberg
Gabriel Nunez
Thorsten Nurnberger
Susanne Nylen
Joshua Obar
Meagan O'Brien
Torsten Ochsenreiter
Ryan O'Connell
Christine O'Connor
Una O'Doherty
Audrey Odom
Kristen Ogden
Marco Oggioni
Tomoaki Ogino
Julia Oh
Helen O'Hare
Dennis Ohman
Päivi Ojala
Hiroaki Okamoto
Bilal Ökmen
Kerry Oliver
Martin Olivier
Ken Olson
Michal Olszewski
Teresa Omeara
Akira Ono
Eng Eong Ooi
Susan Orloff
David Ornelles
Kim Orth
Maghnus O'Seaghdha
Mike Osta
Mario Ostrowski
Melanie Ott
Dong-Yun Ouyang
Julie Overbaugh
Anna Overby
Annette Oxenius
Michelle Ozbun
Yoshihisa Ozoe
Slobodan Paessler
Antonio Pagan
Israel Pagán
Joseph Pagano
Cara Pager
Savita Pahwa
Mirko Paiardini
Gustavo Palacios
Massimo Palmarini
Ann Palmenberg
Sarah Palmer
Kelli Palmer
Marie Pancera
John Panepinto
Giuseppe Pantaleo
Ralph Pantophlet
Nina Papavasiliou
Dimitrios Paraskevis
Piero Parchi
Juan Paredes
Leslie Parent
Daniel Paris
Joanna Parish
Eun Jeong Park
John Parker
Dane Parker
William Parks

Gabriel Parra
Colin Parrish
Tristram Parslow
Marcela Pasetti
Vinay Pathak
Shibani Pati
James Paton
Jean Patterson
James Paulson
Silke Paust
Susan Payne
Amy Pedersen
Joao Pedra
Jorge Pedrosa
Richard Peek
Mark Peeples
Olve Peersen
Michiel Pegtel
Vladimir Pelicic
Jerry Pelletier
Pablo Penaloza
Bennett Penn
Caitlin Pepperell
Mercio Pereiraperrin
Alan Perelson
Rushika Perera
John Perfect
Stanley Perlman
Sallie Permar
George Perry
V. Hugh Perry
Everett Pesci
Nathan Peters
Thomas Peters
Bjoern Peters
Brian Peters
Christine Petersen
Michael Petris
Vasileios Petrou
Gregg Pettis
Susanne Pfeifer
Julie Pfeiffer
Jennifer Philips
Gerald Pier
Susan Pierce
Thomas Pietschmann
David Pigott
Shiv Pillai
Ron Pinhasi
Mariana Pinho
Abraham Pinter
Claudine Pique
Laura Pirisinu
Vicente Planelles
Paul Planet
Oliver Planz
Richard Plemper
Alexander Pletnev
Hidde Ploegh
Alexander Ploss
Stanley Plotkin
Maurizio Pocchiari
Eric Poeschla
Stefan Pöhlmann
Hendrik Poinar
Michael Pollastri
Stephen Polyak
Margarita Pons-Salort
Art Poon
Michel Popoff
Travis Porco
Owen Pornillos
Daniel Portnoy
Jennifer Potts
Michael Povelones
Ann Powers
Rafael Prados-Rosales
Clarissa Prazeres da Costa
Peter Preiser
Roger Preston
David Price
Sean Prigge
Richard Proctor
Anna Protasio
Reeta Prusty Rao
Read Pukkila-Worley

Nathan Pumplin
Georgiana Purdy
Todd Purves
Dohun Pyeon
Alexander Pym
Wei Qian
Jianming Qiu
Feng Qu
Enrique Quesada-Moraga
Valerie Quesniaux
Frederick Quinn
Janet Quinn
Gabriel Rabinovich
Sheli Radoshitzky
Manuela Raffatellu
Jayna Raghwani
Tracy Raivio
Lakshmi Rajagopal
Daniela Rajao
Murugesan Rajaram
Ricardo Rajsbaum
Aurélie Rakotondrafara
Glenn Rall
Katherine Ralston
Sanjay Ram
Gordon Ramage
Holly Ramage
Lalita Ramakrishnan
Jose Ramirez
Jc Ramos
Stefanie Ranf
A. L. N. Rao
Zihe Rao
Jay Rappaport
Chad Rappleye
Rino Rappuoli
Thomas Rasmussen
Phil Rather
Oliver Ratmann
Lee Ratner
Bsrbel Raupach
John Rawls
Ranjit Ray
Stuart Ray
Nick Read
Laurie Read
Fernando Real
Colin Reardon
Meghan Rebuli
Mathias Reddehase
Vijay Reddy
Sarah Reece
Douglas Reed
Michael Reed
R. Keith Reeves
Matthew Reeves
Juan Reguera
Barbara Rehermann
Alan Rein
Barbara Reinhold-Hurek
Michael Reiskind
Jyothi Rengarajan
Michelle Reniere
Rolf Renne
Jesús Requena
Todd Reynolds
Kyu Rhee
Bergmann Ribeiro
David Ribet
Stephen Rice
Andrew Rice
Freddie-Jeanne Richard
Dave Richard
Jack Richards
Anthony Richardson
Leif Richardson
Martin Richer
Douglas Richman
Jeff Riffel
James Riley
Steven Riley
Guus Rimmelzwaan
Cristina Risco
Amariliz Rivera
Fabian Rivera-Chavez
Nadia Roan

Michael Robek
Marjorie Robert-Guroff
Alan Robertson
Derrick Robinson
Leanne Robinson
Richard Robinson
Rosemary Rochford
Barry Rockx
Richard Roden
Isabel Roditi
Ute Roemling
George Rohrmann
Richard Roller
Danilo Roman-Campos
Roy Roop II
Marilyn Roossinck
Michael Root
Rebecca Rose
Ilan Rosenshine
John Rossi
Monica Roth
Stefan Rothenburg
Carla Rothlin
Brice Rotureau
Philippe Roumagnac
Namita Rout
Igor Rouzine
Sarah Rowland-Jones
Chad Roy
Polly Roy
Dennis Rubbenstroth
Brian Rudd
Thomas Rudel
Gloria Rudenko
Kendra Rumbaugh
Jan Rupp
Colin Russell
Charles Russell
David Russell
Ryan Russell
Brent Ryckman
Michelle Ryndak
Wang-Shick Ryu
Juliana Sá
Robert Sabatini
Jonah Sacha
Ruxana Sadikot
Jeroen Saeij
Manish Sagar
Abhik Saha
Neeraj Saini
Mineki Saito
Takeshi Saito
Marco Salemi
Christian Salesse
Sara Salinas
Jeffery Sample
Clare Sample
John Samuelson
Veronica Sanchez
Andrea Sanchez-Vallet
Rogier Sanders
Helene Sanfacon
Dominique Sanglard
Rafael Sanjuán
Teruo Sano
Gilberto Santiago
Erica Saphire
Panagiotis Sapountzis
Martin Sapp
Stefan Sarafianos
Saumendra Sarkar
Dibyendu Sarkar
Peter Sarnow
Kei Sato
Quentin Sattentau
Michael Sattler
Karin Sauer
John-Demian Sauer
Diane Saunders
Alexei Savchenko
Stephen Saville
Lorenzo Savioli
Nancy Sawtell
Charles Scanga
Marcel Schaaf

Heiner Schaal
Luis Schang
Christopher Schardl
Christopher Scharer
Hermann Schatzl
Dirk-Jan Scheffers
Claus Scheidereit
Silke Schelenz
Mario Schelhaas
Mark Schembri
Jeffrey Schertzer
William Schief
Dieter Schifferli
John Schiller
Joerg Schlaak
Mark Schleiss
Larry Schlesinger
Christian Schlieker
Herbert Schmidt
John Schoggins
Herman Scholthof
Robert Schooley
Jeffrey Schorey
Tony Schountz
Hinrich Schulenburg
Stacey Schultz-Cherry
Thomas Schulz
Michael Schurr
Martin Schuster
Olivier Schwartz
Roger Sciammas
Phillip Scott
James Scott-Browne
Gavin Screaton
Peter Sebo
Julie Segre
Rafick Sekaly
Bert Semler
Oliver Semmes
Ruth Serra-Moreno
J. Seshu
Peter Setlow
William Shafer
Manish Shah
Feng Shao
Shayan Sharif
Neelam Sharma-Walia
Paul Sharp
Brian Shaw
George Shaw
Dmitry Shayakhmetov
Noreen Sheehy
Samuel Shelburne III
Wei Shen
Aimee Shen
Li Shengben
Donald Sheppard
Alan Sher
Nathan Sherer
Hai Ning Shi
Pei-Yong Shi
Chiaho Shih
Shin-Ru Shih
Michael Shiloh
Hiroyuki Shimizu
Sunny Shin
Masaki Shintani
Ken Shirasu
Mimi Shirasu-Hiza
Joanna Shisler
Maya Shmulevitz
Sourya Shrestha
Deepak Shukla
Stewart Shuman
L. David Sibley
Shahid Siddique
Inga Siden-Kiamos
Paul Sigala
Christina Sigurdson
Puran Sijwali
Robert Siliciano
Ricardo Silvestre
Olivier Silvie
Graham Simmons
Sanford Simon
Anthony Sinai

Alison Sinclair
John Sinclair
Pradeep Singh
Upinder Singh
Photini Sinnis
Mark Siracusa
Albert Siryaporn
Rebecca Skalsky
Jacek Skowronski
Betty Slagle
James Slauch
Barry Slobedman
Pamela Small
Anna Smed-Sörensen
James Smiley
Darci Smith
Everett Smith
Terry Smith
Gregory Smith
Janet Smith
S. Abigail Smith
Christopher Snyder
Milena Soares
Beate Sodeik
Evgeni Sokurenko
Thierry Soldati
Jay Solnick
Peter Solomon
Ralf Sommer
Hokyoung Son
Justin Sonnenburg
Gabriele Sorci
Joseph Sorg
Claudio Soto
Hugo Soudeyns
Gerald Spaeth
P.D. Spanu
Roberto Speck
Brad Spellberg
Juliet Spencer
Vanessa Sperandio
Katherine Spindler
Gary Splitter
Shiranee Sriskandan
Katryn Stacey
Gary Stacey
Simona Stäger
Christopher Staiger
Henry Staines
Bashar Staitieh
Jason Stajich
Thomas Stamminger
Theodore Standiford
Kenneth Stapleford
Jack Stapleton
Michael Starnbach
John Steel
Olivia Steele-Mortimer
Thilo Stehle
Daniel Stein
David Steinhauer
Eike Steinmann
Joan Steitz
Lorenzo Stella
Steffen Stenger
Robin Stephens
Ioannis Stergiopoulos
Noam Stern-Ginossar
Silke Stertz
Torsten Sterzenbach
Nigel Stevenson
Adrie Steyn
Daniel Stieh
Rinke Stienstra
Timothy Stinear
Monique Stins
Peter Stockley
Daniel Storm
Klaus Strebel
Daniel Streblow
Richard Strugnell
Patrick Stuart
Frank Stubenrauch
Jason Stumhofer
Lishan Su
Carlos Subauste

Agathe Subtil
Andreas Suhrbier
Nancy Sullivan
Christopher Sullivan
William Sullivan Jr.
Yi Sun
Ren Sun
Joseph Sun
Wenxian Sun
Maarit Suomalainen
M. Suresh
Witold Surewicz
Mehul Suthar
Colin Sutherland
Troy Sutton
Phil Sutton
Nobuhiro Suzuki
Catharina Svanborg
Staffan Svard
Yuri Svitkin
Matthew Sweet
Gulam Syed
Moriah Szpara
Fabienne Tacchini-Cottier
Tamar Hamosh Taddei
Yutaka Tagaya
Andrew Tai
Elia Taitwojno
Akifumi Takaori-Kondo
Makoto Takeda
Nicholas Talbot
Vincent Tam
Connie Tam
Rita Tamayo
Gültekin Tamgüney
Tomohiko Tamura
Ming Tan
Hengli Tang
Hua Tang
Wei-Hua Tang
Kenzaburo Tani
Herbert Tanowitz
Yizhi Tao
Caroline Tapparel
Vera Tarakanova
Alexander Tarr
Ann Tate
Ying Taur
Norbert Tautz
Terrie Taylor
Graham Taylor
Geraldine Taylor
Ian Taylor
John Teijaro
Mauro Teixeira
Luis Teixeira
Amalio Telenti
Lesly Temesvari
Italo Tempera
Steven Templeton
Jason Tennessen
Mark Tepfer
Mauricio Terebiznik
Cox Terhorst
Scott Terhune
Hayashi Tetssuya
Waihong Tham
Dimitris Thanos
Gael Thebaud
Andrea Thoma-Kress
Matthew Thomas
Emmanuel Thomas
Nikhil Thomas
Margot Thome
Richard Thompson
Russell Thomson
Hans Thordal-Christensen
Scott Tibbetts
Richard Timms
Toru Tobe
David Tobin
Kostas Tokatlidis
Georgia Tomaras
Fiona Tomley
Massimo Tommasino
Keizo Tomonaga

Shuping Tong
Christopher Tonkin
David Topham
Tuna Toptan
Jordi Torrelles
Victor Torres
Jose Tort
Baldwyn Torto
Zsolt Toth
Jennifer Totonchy
Jean-Nicolas Tournier
Paula Traktman
Leonardo Travassos
Ana Traven
Beth Traxler
Steven Triezenberg
William Trimble
Lindsay Triplett
Ralph Tripp
Emily Troemel
Sai Wah Tsao
David Tscharke
Boo Shan Tseng
Ikuo Tsunoda
Zhijian Tu
Sharof Tugizov
Mick Tuite
Cagla Tukel
Terrence Tumpey
Joanne Turner
Heth R. Turnquist
Rodney Tweten
Matthew Tyska
Juan Ugalde
Glen Ulett
Robert Unckless
Susan Uprichard
Jason Upton
Kevin Urdahl
Edward Usherwood
Jude Uzonna
Vito Valiante
Bruce Vallance
Adrian Valli
Miguel Valvano
Frank van de Veerdonk
Adrianus van der Velden
Giel van Dooren
Koenraad Van Doorslaer
Linda van Dyk
Peter Van Esse
Jan van Kan
Julia Van Kessel
Frank van Kuppeveld
Rene van Lier
Carine Van Lint
Mark van Raaij
Nick Van Reet
Ronald Van Rij
Koen Van Rompay
Jos van Strijp
Wesley Van Voorhis
Gilles van Wezel
Linos Vandekerckhove
Thomas Vanderford
Brian VanderVen
Ramakrishna Vankayalapati
Edouard Vannier
Mariana Varela
Steven Varga
José Ronnie Vasconcelos
Ashley Vaughan
Sue Vaughan
Andres Vazquez-Torres
Jan-Willem Veening
David Veesler
Dominique Velin
Eric Verdin
Georges Verjans
Subhash Verma
Muriel Viaud
Cecile Viboud
Jorge Vidal
Leda Vieira
Gayathri Vijayakumar
Dhanasekaran Vijaykrishna

Andreas Vilcinskas
Didier Vilette
Fernando Villalta
Amy Vincent
Joseph Vinetz
Mark Viney
Kerstin Voelz
Jörg Vogel
Thomas von Hahn
Veronika von Messling
Alice Vrielink
Cuong Vuong
Jatin Vyas
Slavena Vylkova
Andreas Wack
Stephen Waggoner
Ralf Wagner
Samuel Wagner
Matthew Waldor
Mark Walker
Nicholas Wallace
Ross Waller
Pegine Walrad
Patrick Walsh
Derek Walsh
Hongquan Wan
Hans Wandall
Wei-Kung Wang
Xianbing Wang
Zonghua Wang
Guo-Liang Wang
Tony Wang
Jianwei Wang
Xiao-Wei Wang
Yue Wang
Nian Wang
Chen Wang
Xian-Bing Wang
Yan-Yi Wang
Brian Ward
Andrew Ward
Honorine Ward
Matthew Wargo
Digby Warner
Michael Wassenegger
Toshiki Watanabe
Andrew Waters
David Watkins
Joel Watts
Tania Watts
Sing Sing Way
Nathaniel Weathington
Richard Webby
Alexander Weber
Friedemann Weber
K. Scott Weber
Heiner Wedemeyer
Stefan Weger
Taiyun Wei
Christopher Weidenmaier
Leor Weinberger
Daniel Weinberger
Jeffrey Weiser
Susan Weiss
Louis Weiss
Matthew Weitzman
Matthew Welch
Kuo-Feng Weng
Wolfgang Weninger
Silja Wessler
Michelle West
Susan Westmoreland
Dawn Wetzel
Robert Wheeler
Robert White
Michael White
Frank White
Judith White
K. Andrew White
Bradley White
Timothy Whitehead
Malcolm Whiteway
Anna Whitfield
Steven Whitham
Jason Whitmire
Paul Whitney

John Whitney
James Whitney
Gary Whittaker
J. Lindsay Whitton
Lüder Wiebusch
Philipp Wiemann
Brian Wigdahl
Craig Wilen
Clayton Wiley
Steven Wilhelm
Stephan Wilkens
Robert Wilkinson
Angela Wilks
Kenneth Williams
Carolyn Williamson
Kim Williamson
Valerie Williamson
Mark Wills
Shona Wilson
Brenda Wilson
Patrick Wilson
Angus Wilson
David Wilson
Richard Wilson
Mary Wilson
Sam Wilson
Jeffrey Wilusz
William Wimley
Sebastian Winter
Walter Witke
Christiane Wobus
Harald Wodrich
Elizabeth Wohlfert
Jason Wojcechowskyj
Dana Wolf
Steven Wolinsky
Thomas Wolpert
Adrian Wolstenholme
Christiane Wolz
Kyoung-Jae Won
Sook-San Wong
Joseph Wong
Wilson Wong
Patrick Woo
Charles Wood
Stephen Woodward
Joshua Woodward
R. Wooten
Floyd Wormley Jr.
Jens Wrammert
Terry Wright
Peter Wright
Lydia Wroblewski
Ting-Ting Wu
Reen Wu
Qingfa Wu
Marcel Wuethrich
Felix Wussow
Todd Wyatt
Richard Wyatt
Kristine Wylie
Karina Xavier
Ningshao Xia
Yan Xiang
Bingyan Xie
Qi Xie
Xiufang Xin
Zhou Xing
Wei Xu
Chaoyang Xue
Timothy Yahr
Yoshio Yamaoka
Yohei Yamauchi
Nan Yan
X. Frank Yang
Xinzhen Yang
Liang Yang
George Yap
Robert Yarchoan
Felix Yarovinsky
Jian Ye
Gongyin Ye
Sheng Ye
Michael Yeaman
Hasan Yesilkaya
Jonathan Yewdell

Minkyung Yi
Jeffrey Yoder
Puangrat Yongvanit
Keiko Yoshioka
Jianxin You
Paul Young
Lawrence Young
Benjamin Youngblood
Jacob Yount
Sung-Liang Yu
Xiao-Fang Yu
Yan Yuan
Jose Yuste
Mark Zabel
Dietmar Zaiss
Allan Zajac
Gianluigi Zanusso
Colby Zaph
Michael Zasloff
Gianguglielmo Zehender
Lirong Zeng
Leigh Zerboni
Qijing Zhang
Xuming Zhang
Zhengguang Zhang
Lili Zhang
Zhonghui Zhang
Junjie Zhang
Bo Zhao
Jun Zhao
Binlian Zheng
Chunfu Zheng
Zhi-Ming Zheng
Jin Zhong
Pei Zhou
Bin Zhou
Shuntai Zhou
Jinfang Zhu
Ying Zhu
Hua Zhu
Fanxiu Zhu
Joseph Ziegelbauer
Véronique Ziegler-Graff
Dan Zilberstein
Sara Zimmer
Albert Zink
Adam Zlotnick
Wen-Quan Zou
Chiara Zurzolo
Michael Zwick
